# Whole lung lavage combined with Granulocyte-macrophage colony stimulating factor inhalation for an adult case of refractory pulmonary alveolar proteinosis

**DOI:** 10.1186/1471-2466-14-87

**Published:** 2014-05-19

**Authors:** Hong yan Yu, Xue feng Sun, Yan xun Wang, Zuo jun Xu, Hui Huang

**Affiliations:** 1 Department of Respiratory Medicine, Peking Union Medical College Hospital, Chinese Academy of Medical Sciences & Peking Union Medical College, #1 Shuaifuyuan Street, Dongcheng District, 100730 Beijing, China

**Keywords:** Refractory pulmonary alveolar proteinosis (PAP), GM-CSF inhalation, Plasmapheresis, Whole lung lavage (WLL), Combination therapy

## Abstract

**Background:**

Whole-lung lavage (WLL) is classically the first-line treatment for symptomatic pulmonary alveolar proteinosis (PAP). However, some patients require multiple WLLs because of refractory nature of their PAP. In this instance, these patients may benefit from new treatment regimens, and new therapies should be tried for these patients.

**Case presentation:**

We describe a 47-year-old Chinese woman who was confidently diagnosed with pulmonary alveolar proteinosis (PAP) after bronchoalveolar lavage and transbronchial lung biopsy. The patient received four sessions of bilateral whole lung lavage (WLL) and one session of WLL in combination with plasmapheresis, each only producing short-term symptomatic relief. The patient was given a trial of combination therapy, which consisted of WLL and Granulocyte-macrophage colony-stimulating factor (GM-CSF) inhalation. The patient showed a gradual improvement in oxygenation and her daily activity, as well as a dramatic improvement in her pulmonary CT examination.

**Conclusion:**

Bilateral WLL, in combination with GM-CSF inhalation, may be an effective treatment option for severe refractory PAP.

## Background

Pulmonary alveolar proteinosis (PAP) is a rare but treatable disease that is characterized by the accumulation of a periodic acid Schiff (PAS) positive lipoproteinaceous substance in the alveoli. Idiopathic PAP (iPAP), also known as “autoimmune pulmonary alveolar proteinosis”, makes up 90% of all described cases [1]. Whole lung lavage (WLL) is the most common first-line treatment for symptomatic iPAP. However, significant proportions of patients require multiple WLLs, or do not respond to WLL. Many alternatives to WLL, such as GM-CSF supplementation and plasmapheresis, have been introduced. However, the treatment of severe refractory cases still remains a major challenge for clinicians in which combination therapy instead of monotherapy maybe a better choice. Here we describe an adult case of severe refractory PAP successfully treated with WLL in combination with sequential GM-CSF inhalation therapy.

## Case presentation

**Figure 1:**
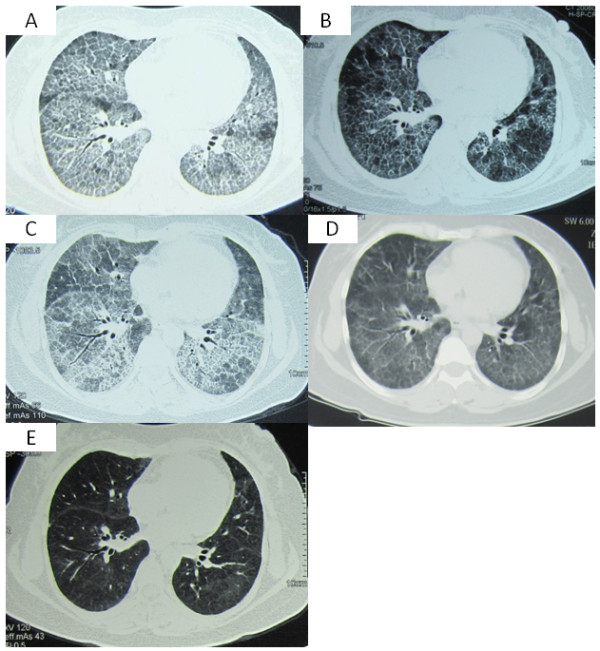
**Series of her chest CT. A**. The patient’s chest CT at the time of diagnosis of PAP. Her chest CT on Sep 10^th^ 2010 showed geographic ground-glass opacities combined with interlobular septal thickening (crazy paving) in both lungs. **B**. The patient’s chest CT after one month of the first WLL. On Oct 20^th^ 2010, about one month after the initial WLL, her repeated chest CT showed that the ground-glass opacities were diminished in both lungs. **C**. The patient’s chest CT during the the fourth refractory episode of PAP. On May 26^th^ 2012, the patient’s chest CT showed increased crazy paving in both lungs. **D**. The patient’s chest CT after the combination of WLL and 5 weeks of inhalation of GM-CSF. Her chest CT on Jul 8^th^ 2012 showed diminished shadows in both lungs. **E**. The patient’s chest CT after the combination of WLL and 15 weeks of inhalation of GM-CSF. There was significant improvement in her repeated chest CT on Sep 17^th^ 2012.

A 47 year old HAN female, with no past medical history, presented to our clinic complaining of shortness of breath and a productive cough with white sputum. After thorough investigation, she was diagnosed with pulmonary alveolar proteinosis. High resolution CT scan (HRCT) of her chest demonstrated the classic PAP features of crazy paving (Figure 1A). Her bronchoalveolar lavage fluid (BALF) was milky in appearance, and periodic acid-Schiff (PAS) staining of the lavage fluid revealed accumulation of an excessive pinkish protein-like substance. Hematoxylin and eosin staining of the patient’s transbronchial lung biopsy (TBLB) showed multiple alveolar spaces and bronchioles filled with a granular acidophilic acellular material, which stained bright pink with PAS. Her room air arterial blood gas analysis was consistent with type 1 respiratory failure, but could be corrected with oxygen via nasal cannula (NC) at 2 L/min. Her blood chemistry panel was normal, with a LDH of 195 U/L. Pulmonary function testing revealed a restrictive pattern with impaired diffusion: FVC 2.14 L/71.4% predicted, FEV1: 1.95 L/75.8% predicted, FEV1/FVC 90.94%, and DLco 3.19 mmol/min/kPa/39.5% predicted, respectively.

The first bilateral sequential WLL was performed under general anesthesia in September of 2010, which improved her symptoms and her P_(A-a)_O_2_ substantially for about 1 year. However, in October of 2011, her cough and exertional dyspnea returned, with a room air PaO_2_ of 42 mmHg. Supplemental oxygen was given at 7 L/min via NC to help correct her severe hypoxemia. At this time a second bilateral WLL was performed, which did not improve her symptoms or hypoxia. Thus, a third WLL was to be repeated in one month (November 2011). Three months later, in February of 2012, the patient had a third flare of PAP which was thought to have been triggered by the common cold. Considering the limited efficacy of WLL therapy, plasmapheresis was considered as a possible alternative treatment. She then received bilateral WLL followed by 5 sessions of plasmapheresis over 2 weeks, at an exchange volume of 2.5 L. Her clinical symptoms and radiological manifestations improved temporally. In May of 2012, her PAP relapsed again (Figure 1C), and O_2_ 8-9 L/min via NC was required to maintain adequate oxygenation. With the approval from the Ethics Committee of our hospital, exogenous GM-CSF inhalation was given. Considering that patients treated with GM-CSF usually take time to respond to therapy, we applied GM-CSF inhalation therapy in combination with WLL. The patient received WLL on June 1^st^ 2012 first, and then was started on rhGM-CSF inhalation therapy at a dosage of 150 μg twice daily for 8 days, which was then stopped for 6 days. This cycle was repeated every 2 weeks over the next 3 months. In her follow-up visit in September of 2012, she showed no signs of dyspnea, and had a PaO2 of 68 mmHg on room air, as well as a significant improvement in her chest CT (Figure 1D, E). No substantial adverse events were reported. Another course of GM-CSF inhalation at a dosage of 150 μg was suggested. Again given in cycles of once daily for 8 days, and then every 2 weeks over 6 months. Until now, the patient has had no further flares of her PAP, and was able to take care of herself without difficulty. The patient’s complete course of treatment has been listed in Table 1.

**Table 1:** Treatment of the refractory adult PAP case (listed according to timeline)

**Time**	**Treatment choice**	**Details**	**Patient response**
SEP 16^TH^, 2010	Whole lung lavage	Left lung: 8 L saline flushed in, 7.5 L recycled. Right lung: 9 L saline flushed in, 8.88 L recycled.	Good response, improvement in symptoms and P_A-a_ maintained throughout 1 year
OCT 13^TH^, 2011	Whole lung lavage	Left lung: 5.9 L saline flushed in, 5.68 L recycled. Right lung: 7 L saline flushed in, 6.8 L recycled.	Improved symptoms and P_A-a_;
NOV 16^TH^, 2011	Whole lung lavage	Left lung: 8.0 L saline flushed in, 7.7 L recycled. Right lung: 7 L saline flushed in, 6.75 L recycled.	Improved symptoms, P_A-a,_ and pulmonary function; good control of symptoms through 3 months.
FEB 2^ND^, 2012	Whole lung lavage	Left lung: 8.0 L saline flushed in, 7.82 L recycled. Right lung: 8 L saline flushed in, 7.71 L recycled.	Symptoms and hypoxia not improved, still required high volume oxygen support;
FEB 8^th^-20^th^, 2012	5 sessions of plasmapheresis therapy	Exchange volume: 2.5 L, every 2–3 days during two weeks.	
JUNE 1^ST^, 2012	Whole lung lavage	Left lung: 9.0 L saline flushed in, 9.33 L recycled. Right lung: 7.0 L saline flushed in, 6.98 L recycled.	Good symptomatic control, with no hypoxia and no further flare of PAP
JUNE 2^nd^, 2012	GM-CSF inhalation	At a dosage of 150 μg, twice daily from day 1 to day 8, with inhalation stopped from day 9 to day 14, every 2 weeks for 3 months;	
Sep, 2012	GM-CSF inhalation	At a dosage of 150 μg, once daily from day 1 to day 8, every 2 weeks for 6 month;	

WLL, whole lung lavage; PAP, pulmonary alveolar proteinosis; GM-CSF, Granulocyte-macrophage colony-stimulating factor.

During the WLL, we used aliquots of (600-800) ml.

## Discussion

Multiple treatment options have been introduced into clinical managment of PAP, including WLL, plasmapheresis, GM-CSF supplementation, Rituximab, and lung transplantation. Whole lung lavage, first introduced by Ramirez in 1965 [2], has long been the traditional choice of treatment in PAP, and was the only therapy for many years. Over years of practice and experience with PAP, the techniques regarding WLL have improved, however no consensus has yet been reached. The outcomes and efficacy of WLL has been studied extensively by various centers. A recent study by Campo et al. reported the follow up data of 44 Italian PAP patients treated with WLL from a single center [3]. Over a two-decade period, 31/44 patients required only one WLL to maintain long term symptomatic control, with 13/44 requiring multiple WLLs. Beccaria et al. studied 21 idiopathic PAP patients treated with WLL, with 18/21 requiring only one session of WLL whereas 3/21 underwent multiple WLL for recurrent PAP. In this study, more than 70% of patients remained relapse-free over a 7-year follow up period [4]. Also, Shah et al. shared their experience in treating PAP patients with WLL [5], concluding that in their experience more than 60% of patients had a good response within two sessions of WLL. Less than 15% of these patients required WLL every six months to maintain their functional status, and fewer than 10% were non-responsive. In our case, the patient had a good response for the first WLL. However, multiple sessions of WLLs were subsequently required, each with a shortened duration of effectiveness.

A GM-CSF autoantibody has been identified in PAP patients but not in healthy controls [6], indicating a potential pathologic role in PAP pathogenesis. More interestingly, Sakagami et al. successfully reproduced PAP in nonhuman primates after injecting patient derived GM-CSF autoantibodies into these experimental animals [7,8]. This further suggests a direct role of GM-CSF autoantibodies in the pathogenesis of PAP. Thus it is a reasonable approach to use plasmapheresis to reduce circulating autoantibodies in the treatment of PAP. However, the experience of applying plamapheresis in PAP patients is still quite limited, with data only from case-reports to evaluate its efficacy. Also, there have been multiple conflicting conclusions amongst them. Bonfield et al. [9] reported a PAP patient which was unresponsive to GM-CSF treatment, and while waiting for lung transplantation received 10 sessions of plasmapheresis at an exchange volume of 1.5 L. Over a 2-month period the patient’s anti-GM-CSF titer was lowered, as well as a substantial improvement in their oxygen saturation and chest radiograph. The other case presented by Luisetti et al. [10] however showed a more passive result. After 10 sessions of plasmapheresis (at an exchange volume of 1.5 L) anti-GM-CSF levels were reduced by half. However, there were no improvement of the patient’s symptoms, chest radiographs, or pulmonary function. The interval between repeated WLLs seemed to have been elongated. Here in our case, 5 continuous sessions of plasmapheresis at an exchange volume of 2.5 L had a very small improvement in symptoms and pulmonary function. Plasmapheresis as an alternative treatment in PAP still requires more investigation.

Exogenous GM-CSF supplementation is a possible rescue treatment, and has thus been under extensive investigation. So far two routes of GM-CSF administration have been introduced: inhalation and subcutaneous. In a meta-analysis reviewing the efficacy of GM-CSF in PAP treatment, 5 observational studies (involving 94 cases) were examined, including 3 studies using subcutaneous GM-CSF administration and 2 studies on GM-CSF inhalation [11]. According to this meta-analysis, the response rate of GM-CSF therapy ranged from 43% to 92% in different studies, and the calculated pooled response rate by the random effects model was 58.6% (95% CI, 42.7-72.9). Fever and local erythema at the injection site were the most commonly reported complications. As the alveolar space was deemed as the putative site of the GM-CSF signal disruption, inhalation therapy should have better deposition with lower dosage and less effects on the bone marrow than injection therapy. Although the difference was not statistically significant, the inhalation group had a better response rate than the subcutaneous group (76.5%; 95% CI, 34.5-95.3 vs 48.4%; 95% CI, 33.8-63.3). The relapse rate and incidence of adverse events were also lower in the inhalation group. Thus, GM-CSF inhalation was recommended for our patient. Evidence also shows a slightly better performance of GM-CSF inhalation therapy at a dosage of 250 μg/day, every second week [11]. But, since the only available formulation of GM-CSF in our hospital is 150 ug per dose, we decided to give her GM-CSF at a dosage of 150 ug twice daily from day 1 to day 8, and repeated this every two weeks. It is important to note that in these trials there was usually a lag period of 4 to 12 weeks in the inhaled group prior to showing a clinical response [substantial P_(A-a)_O_2_ improvement (≥10 mm Hg)] [12]. In conclusion, GM-CSF inhalation combined with WLL maybe used to achieve a better outcome in patients with PAP, as is the case in the patient we previously mentioned.

## Conclusion

In conclusion, until now, there have been no studies conducted to evaluate the efficacy of combination therapy for PAP. Only several case reports are available, similar to ours. This case has demonstrated the effectiveness of combination therapy for a refractory PAP case. Combination therapy is primarily indicated, and holds promise, in refractory or unresponsive cases, and because of its scarcity, it is hard to summarize and compare the efficacy of different combinations. For severe refractory PAP cases, we suggest that bilateral WLL combined with sequential GM-CSF supplementation may be an excellent treatment option. However, WLL and plasmapheresis in combination were not as effective in this case. We regret that during our management of this case, due to lack of experience of our clinical laboratory’s ability to quantify the GM-CSF autoantibody, we were unable to test and monitor GM-CSF autoantibody levels during the diagnosis and treatment of this patient.

## Consent

Written informed consent was obtained from the patient for publication of this Case report and any accompanying images. A copy of the written consent is available for review by the Editor-in-Chief of this journal.

## Abbreviations

PAP: Pulmonary alveolar proteinosis; WLL: Whole-lung lavage; GM-CSF: Granulocyte-macrophage colony-stimulating factor; BALF: Bronchoalveolar lavage fluid; PAS: Periodic acid-schiff; TBLB: Transbronchial lung biopsy.

## Competing interests

The authors do not have any competing interests and/or bias with regard to this publication.

## Authors’ contributions

ZJX and HH served as the guarantor of the paper and takes responsibility for the integrity of the work as a whole. HYY and XFS conceived the study and participated in its design and coordination. HYY performed the statistical analysis and drafted the manuscript. XFS and YXW participated in data collection. All authors read and approved the final manuscript.

## Pre-publication history

The pre-publication history for this paper can be accessed here:

http://www.biomedcentral.com/1471-2466/14/87/prepub
